# The cytochrome P450 (CYP) gene superfamily in *Daphnia pulex*

**DOI:** 10.1186/1471-2164-10-169

**Published:** 2009-04-21

**Authors:** William S Baldwin, Peter B Marko, David R Nelson

**Affiliations:** 1 Clemson University, Biological Sciences, Institute of Environmental Toxicology, 509 Westinghouse Road, PO Box 709, Pendleton, SC, USA; 2 Clemson University, Biological Sciences, 132 Long Hall, Clemson, SC, USA; 3 University of Tennessee-Memphis, Department of Molecular Sciences, G01 Molecular Science Building, Memphis, TN, USA

## Abstract

**Background:**

Cytochrome P450s (CYPs) in animals fall into two categories: those that synthesize or metabolize endogenous molecules and those that interact with exogenous chemicals from the diet or the environment. The latter form a critical component of detoxification systems.

**Results:**

Data mining and manual curation of the *Daphnia pulex *genome identified 75 functional CYP genes, and three CYP pseudogenes. These CYPs belong to 4 clans, 13 families, and 19 subfamilies. The CYP 2, 3, 4, and mitochondrial clans are the same four clans found in other sequenced protostome genomes. Comparison of the CYPs from *D. pulex *to the CYPs from insects, vertebrates and sea anemone (*Nematostella vectensis*) show that the CYP2 clan, and to a lesser degree, the CYP4 clan has expanded in *Daphnia pulex*, whereas the CYP3 clan has expanded in insects. However, the expansion of the *Daphnia *CYP2 clan is not as great as the expansion observed in deuterostomes and the nematode *C. elegans*. Mapping of CYP tandem repeat regions demonstrated the unusual expansion of the CYP370 family of the CYP2 clan. The CYP370s are similar to the CYP15s and CYP303s that occur as solo genes in insects, but the CYP370s constitute ~20% of all the CYP genes in *Daphnia pulex*. Lastly, our phylogenetic comparisons provide new insights into the potential origins of otherwise mysterious CYPs such as CYP46 and CYP19 (aromatase).

**Conclusion:**

Overall, the cladoceran, *D. pulex *has a wide range of CYPs with the same clans as insects and nematodes, but with distinct changes in the size and composition of each clan.

## Background

The cytochrome P450s (CYPs) have widespread and diverse functions in animals. CYPs in families 1–4 are critical and often inducible components of the phase I detoxification systems of vertebrates, invertebrates, and plants [1-4]. They are also important in lipid metabolism, including fatty acids, retinoids, eicosanoids, steroids, vitamin D and bile acids, and some are regulated in a sexually dimorphic fashion through endocrine mechanisms [5-8]. In addition, the CYP26 family contributes to maintenance of sharp retinoic acid boundaries between rhombomeres in the developing mammalian embryo and in the eye [9-11]. In insects CYPs are essential to the function of sensory organs such as antennae, where they may be involved in odorant clearance [12].

CYPs are important in the metabolism of and tolerance to anthropogenic chemicals [13] and plant allelochemicals [14,15]. Inducibility, constitutive overexpression, and genomic studies investigating quantitative trait loci (QTL) mapping have demonstrated tolerance or resistance to environmental chemicals due to CYPs [13,16,17]. Furthermore, it has been hypothesized that pesticide sensitivity in honeybee is associated with reduced CYP numbers [18]. In addition, hormone structure is dependent on CYPs [19], and therefore small differences in the hormones utilized (i.e. juvenile hormones, methyl farnesoate) may be dependent on the CYPs available or the timing of expression.

*Daphnia pulex*, commonly known as the waterflea, is the first crustacean to have its genome sequenced. *D. pulex *has a genome of approximately 200 Mb with 31,000 genes . Daphnid species are globally distributed zooplankton, in the order Branchiopoda, suborder Cladocera, and there are several daphnid species, including *Daphnia magna*, *Daphnia pulicaria*, *Daphnia pulex*, and *Ceriodaphnia dubia*. Daphnids are commonly studied zooplankton because of their importance to aquatic ecosystems, ability to contend with environmental challenges, amenability to culture, short life-cycle, and parthenogenic reproduction. Furthermore, daphnids are commonly used in several toxicity tests for multiple applications. Studies on the *Daphnia pulex *genome and specifically the CYPs may provide important insight on how genome and gene expression alterations promote individual and population-level fitness following environmental change.

It has become clear in the genomic era that previous estimates for the number of CYPs in daphnids such as *D. magna *[20] was a gross underestimate, and that most metazoans have a large number of CYP genes. For example, humans have 57, mice have 102, *Stronglyocentrotus purpuratus *(sea urchin) have 120, *Anopheles gambiae *(mosquito) have 106, and *Drosophila melanogaster *have 83 functional CYPs (Additional file 1). In continuation of this P450 research, we are working with the *Daphnia *Genomics Consortium to assemble, annotate, and compare the CYPs in the *Daphnia pulex *genome with several other species. We have also assigned names to all of the CYPs and CYP pseudogenes based on previously published rules using phylogenetic trees and amino acid sequence identity to determine clan, family and subfamily membership [21]. Clans represent the deepest diverging gene clades in the CYP nomenclature. There are ten P450 clans among animals, but only four are present in the protostomes: CYP2, CYP3, CYP4 and mitochondrial. The general web repository for P450 nomenclature and sequence data is . Overall, this work provides a continuation of earlier projects to comprehensively annotate CYPs and determine the putative role of *Daphnia pulex *CYPs in sensory adaptation, sensitivity to toxicants, adaptation to environmental challenges, and as biomarkers of chemical or endocrine stimulation.

## Results and discussion

### Manual Annotation

Manual annotation and curation of the CYPs in *Daphnia pulex *v1.1 draft genome sequence assembly (September, 2006) produced 73 full length P450s and three pseudogenes. In addition, there are two ESTs in the CYP4 clan that were not found in the *D. pulex *genome of the chosen parthenogenic individual, but partial sequences (Cyp4C32, Cyp4AN1) were observed previously in specific *D. pulex *ecotypes [15]. Overall, this number is similar to other CYP genomes (Additional file 1), but slightly lower than the typical invertebrate or insect with the exception of honeybee [22].

**Figure 1:**
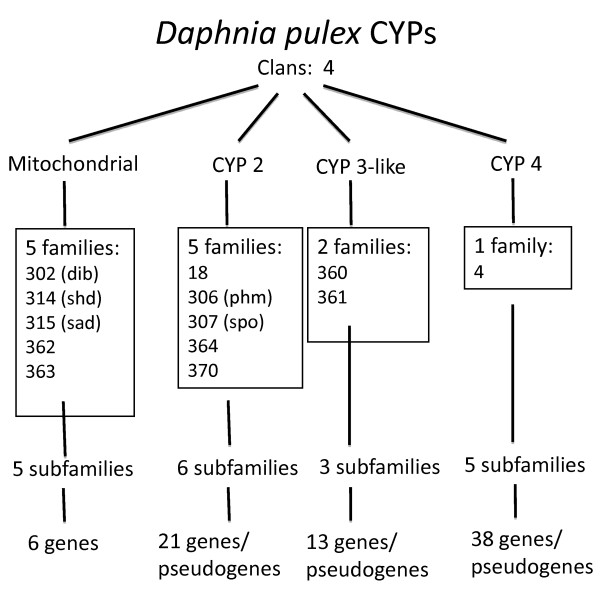
***Daphnia pulex *CYPs are divided into fourclans; mitochondrial, CYP2, CYP3, and CYP4**. The four clans can befurther subdivided into 13 families, 19 subfamilies, and 78 isoforms, including pseudogenes. The numbers represented above include thepseudogenes, of which there is one pseudogene in each of the CYP2, 3, and 4 clans.

**Figure 2:**
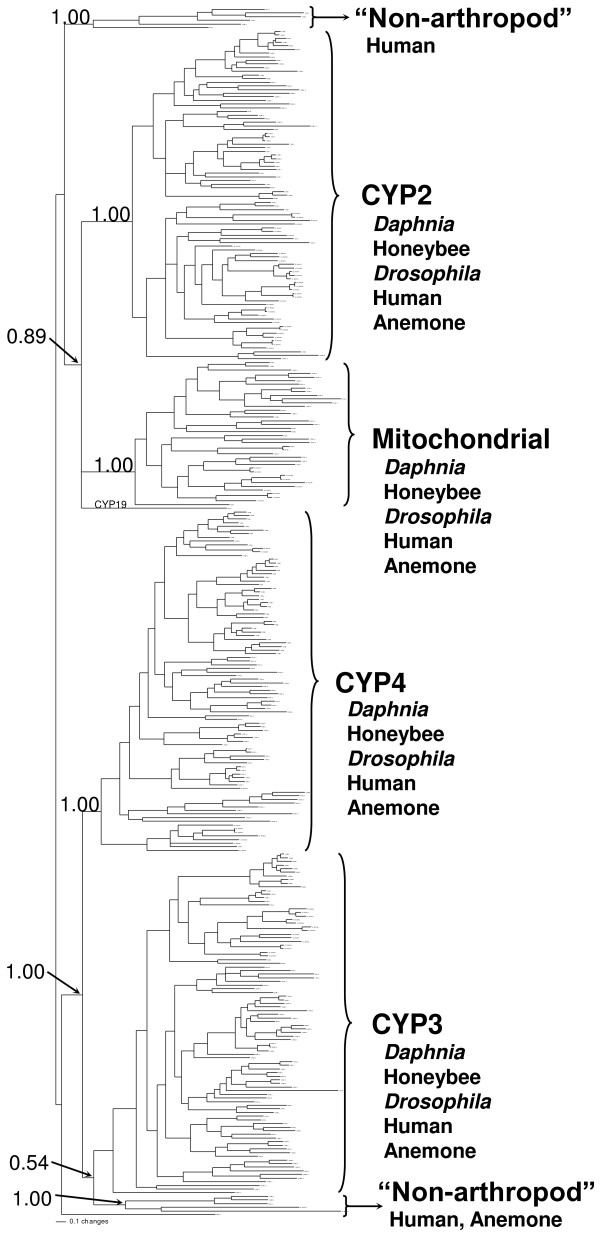
**Phylogenetic relationship of the different CYP clans**. Five CYP genomes were subjected to phylogenetic comparisons using MrBayes. An (H) after the CYP name denotes human sequences, a (B) denotes honeybee sequences, a (M) denotes *Drosophila melanogaster *sequences, CYP names lacking a letter are *D. pulex *sequences, and anemone sequences are noted with their GenBank protein accession numbers (start with XM). Numbers at nodes are posterior probabilities from the Bayesian analysis. Figure 2 is also available in an easily readable, expandable pdf format as a supplementary figure that allows recognition of individual CYP isoforms within each clade (Additional file 2).

The *Daphnia pulex *full length CYPs are divided into four distinct clans; mitochondrial, CYP2, CYP3, and CYP4, the same clans that are found in insects [22]. The four clans can be further subdivided into 13 families, 19 subfamilies, and 75 putatively functional isoforms, and 3 pseudogenes (Fig. 1). The phylogenetic tree in combination with the previously described rules for naming CYPs (40% identity for family designation, 55% identity for subfamily designation) were used to name the different CYPs in *D. pulex *[21]. Six new CYP families were discovered; mitochondrial clan families CYP362 and 363, CYP2 clan families CYP364 and 370, and CYP3 clan families CYP360 and 361 (Fig. 1, 2).

### Phylogenetic tree

Bayesian inference and maximum parsimony yielded nearly identical topologies, so we have presented the tree in which branch lengths are proportional to the amount of change occurring in each lineage (Fig. 2). This tree is also available in a detailed, expandable, readable pdf document as supplementary material (Additional file 2). The Bayesian tree includes posterior probabilities at nodes which reflect the proportion of trees sampled during the search that included each particular node. The sea anemone genome has not been manually curated and therefore the data are subject to potential assembly errors until validated. However, the sea anemone provides an ancient anthozoan class cnidarian, and more importantly a diploblast, to the analysis as the only non-triploblastic species in our phylogenetic tree. The honeybee and fruitfly provide a protostome insect CYP genome for comparison, while the human CYPs provide a deuterostome for comparison to the Branchiopod crustacean, *D. pulex*.

In general, the tree shows six distinct monophyletic clades, all but one supported by posterior probabilities of 1.00. These include the mitochondrial, CYP2, CYP3, and CYP4 clans, and two deep branches that do not include any arthropod CYPs. The non-arthropod lineages are nearly deuterostome exclusive and would be if not for the two anemone CYPs that show their closest relationships to CYP26. These anemone CYPs found nested within a vertebrate lineage indicate that a CYP26-like ancestor may have existed in cnidarians but was lost in protostomes. Similar inferences concerning CYP loss in protostomes have already been made about CYP51, CYP20 and possibly CYP7. The history of CYP20 in protostomes may be complex since an ortholog has been detected in the annelid leech *Haementeria depressa *(CN807321). In addition, CYP19 (aromatase) clusters with the CYP2 and mitochondrial clans, and CYP46 and related sea anemone CYPs are part of a sister clade to the CYP4 clan (Additional file 2).

Overall, the tree demonstrates that the four major clans found in insects (mitochondrial, CYP2, 3, 4) encompass all of the CYPs in *D. pulex*. An Excel table of each of the CYP genes and pseudogenes found in the *Daphnia pulex *genome, their nucleotide and amino acid sequences, and links to their scaffold position is available as supplementary material (Additional file 3).

The mitochondrial clan of *D. pulex *contains six members in five families and five subfamilies. Three of the members are highly conserved Halloween genes involved in ecdysone synthesis [19,23]. Specifically, *disembodied *(Cyp302A1; *dib*), *shade *(Cyp314A1, *shd*), and *shadow *(Cyp315A1; *sad*) are all mitochondrial CYPs involved in the last three steps of 20-hydroxyecdysone (20-HE) synthesis. Cyp314A1 is the CYP required for the conversion of ecdysone to its active form, 20-HE [19,23]. The other three CYPs are divided into two new families CYP362 (Cyp362A1, Cyp362A2) and Cyp363A1, indicating that these CYPs are not as well conserved and probably have taken on new roles. A tree of the mitochondrial CYP clan is available as supplementary material (Additional file 4).

**Figure 3:**
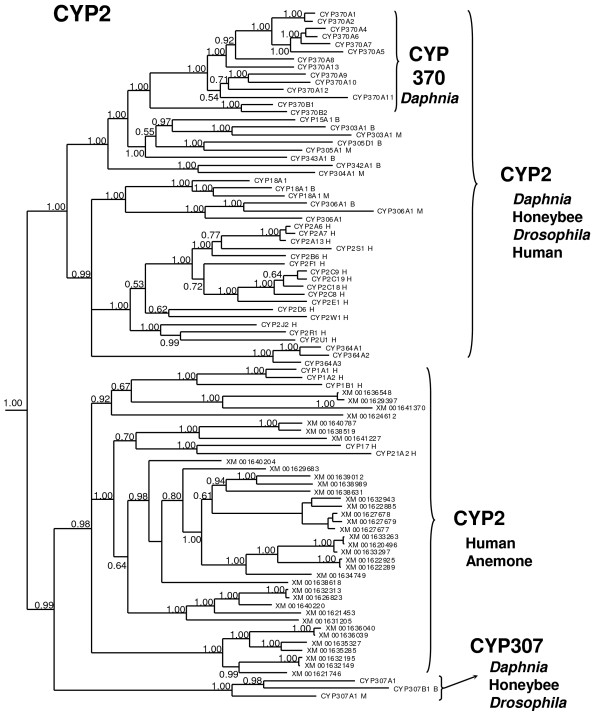
**Phylogenetic relationship of the different CYP2 clan members**. Three CYP2 clades are observed; two that contain arthropod CYP2 members and one that does not. In addition, the CYP2 clan is expanded in *Daphnia pulex *because of expansion of the Cyp370 family. An (H) after the CYP name denotes human sequences, a (B) denotes honeybee sequences, a (M) denotes fruitfly sequences, CYP names lacking a letter are *D. pulex *sequences, and anemone sequences are noted with their GenBank protein accession numbers (start with XM). Numbers at nodes are posterior probabilities.

The CYP2 clan of *D. pulex *contains 21 members, including one pseudogene (not shown in Fig. 2, 3), separated into five distinct families (Cyp18, 306, 307, 364, 370) and six subfamilies. Two of the genes, *phantom *(Cyp306A1; phm) and *spook *(Cyp307A1; *spo*) are conserved Halloween genes involved in the early stages of ecdysone synthesis [19,24]. The exact role of Cyp307A1 in ecdysone biosynthesis is unknown, but appears to be part of the "Black box" of reactions involved in the earlier steps of ecdysone synthesis from cholesterol. *Daphnia pulex *only contains one Cyp307 gene similar to sequenced lepidopterans, but different than *Drosophila *that use two Cyp307 genes at different stages of their lifecycle [24,25]. Interestingly, the Cyp307 genes are a sister group to the non-arthropod CYP2 members (Fig. 3).

The other three CYP2-clan families in *D. pulex *(Cyp18, 364, 370) are divided into four subfamilies and contain 19 genes. Interestingly, a sister-group in the CYP2 clan contains no arthropod CYPs. This group primarily contains anemone CYPs, but also contains CYP17, CYP21, and the CYP1 family members inducible by chlorinated hydrocarbons in vertebrates [26](Fig. 3; also available as Additional file 5). The Cyp370 family is the largest of the CYP2-clan families, containing 15 members with 13 members in the 370A subfamily and 2 members in the 370B subfamily (Cyp370B1,2). There is also one pseudogene in the Cyp370A subfamily, Cyp370A3P.

The Daphnia CYP370 family is greatly expanded relative to the single gene CYP15 and CYP303 families in insects it most closely resembles. Cyp15A1 is a regio- and stereo-specific epoxidase critical in the formation of juvenile hormone III (JH III) from methyl farnesoate in the corpora allata of the cockroach [27]. However, methyl farnesoate, a juvenile hormone precursor, is considered the major terpenoid hormone in crustaceans [28]; therefore, a methyl farnesoate epoxidase is unnecessary and it is unlikely that the CYP370A and CYP370B subfamily members specifically perform this function. The role of the Cyp370 family in *Daphnia *is currently unknown. Cyp18 and Cyp364 are both close relatives of Cyp306 (*phantom*), suggesting potential involvement in ecdysone synthesis or catabolism (Fig. 3). The Cyp364 family is a new family that contains three genes (Cyp364A1,2,3). Cyp18, which is also found in insects, is induced by 20-HE in *Drosophila *[29].

The CYP3 clan consists of numerous CYPs involved in detoxification of xenobiotics and endobiotics [30-33]. Some CYP3 clan members are inducible by hormones such as progesterone [34] and ecdysone [17], and are responsible for the metabolism and elimination of steroid hormones in vertebrates [20,35,36]. Although the posterior probability at the base of the CYP3 clan is low (0.54), this only reflects the uncertainly of the position of the first two *Drosophila *lineages at the base of the CYP3 clan. The CYP3 clade is strongly supported when *Drosophila *is not included in the analysis (1.00). In *Daphnia pulex*, the CYP3 clan contains 12 genes and one pseudogene, arranged into two new families (Cyp360, Cyp361) and three subfamilies. Eleven of these thirteen genes are in the Cyp360A subfamily leaving just Cyp361A1 and Cyp361B1 outside this subfamily in the Cyp3 clan of *D. pulex*. The closest relatives of the Cyp360 subfamily in the tree are the Cyp6 and Cyp9 subfamily members of insects involved in endobiotic and xenobiotic metabolism and detoxification. Similarly, the closest relatives of the two Cyp361 subfamily members are the anemone CYP3-like group, and the human CYP3A and CYP5A subfamily members involved in detoxification and thromboxane A_2 _biosynthesis. In general, the CYP3 clan in insect species has more CYPs than the *Daphnia pulex *CYP3 clan. Most CYP families in insects have only a few members, but the CYP6AS subfamily has 18 members, 37.5% of the honeybee P450s, and there are 35 members of the CYP3 clan distributed between seven families making up 42% of the *D. melanogaster *P450s. The CYP3 clan in insects, and in particular the CYP6AS subfamily in honeybee was recruited for major gene expansion as were the CYP360 and CYP370 families in *D. pulex*, and to a lesser degree the CYP4C subfamily. A tree of the CYP3 clan is available as an additional file (Additional file 6).

The CYP4 clan, which is the sister-group to a clade containing the CYP3 clan plus one of the two "non-arthropod" clans, consists of 38 members all in the same family (Cyp4) and arranged into five subfamilies (Cyp4C, Cyp4AN, Cyp4AP, Cyp4BX, Cyp4BY) with 4–10 members in each subfamily (Additional file 7). There is also a pseudogene in the Cyp4C subfamily. Two members of the Cyp4 family were not observed in the *Daphnia pulex *genome v1.1 draft genome sequence assembly (September, 2006), but were cloned by degenerative PCR in a previous study in which nine CYP4 members were partially cloned [15]. The two absent CYPs, Cyp4C32 (95% identical to 4C34v1; 89% to 4C34v2 from the *Daphnia pulex *genome) and Cyp4AN1 (92% to 4AN2v1; 96% identical to CYP4AN2v2 from the *Daphnia pulex *genome), are available on GenBank (BQ703381 and BQ703379, respectively). The *D. pulex *genome sequence coverage was 8.7×, therefore our inability to find these two CYPs may be due to the known gaps in the genome assembly. It is also possible that these two genes were deleted from the Daphnia Genomics Consortium's chosen parthenogenic *D. pulex *or "chosen one" due to strain differences.

The Cyp4 members are considered the least studied of the CYP clans in insects [22] and are involved in fatty acid metabolism, including inflammatory arachidonic acid metabolites, and xenobiotic metabolism in mammals [37]. Some CYP4 members may be involved in sensory perception in insects as they are found in the antenna [38]. A Cyp4c member is also involved in the biosynthesis of juvenile hormone, and another is inducible by hypertrehalosemic hormone, a key hormone in arthropod carbohydrate metabolism [39]. Furthermore, some Cyp4 members are down-regulated by ecdysteroids [40], indicating that Cyp4 members may play a key role in sensory and hormonal functions in *D. pulex*.

Primarily CYP3, but some CYP4 and mitochondrial clan members have been associated with resistance to pesticides [17,30,41-44]. Several Cyp3 clan members, such as Cyp6g1 and Cyp6a5 are associated with DDT or pyrethroid resistance, respectively [41,44]. Cyp4D10 and other CYP4 members in *Drosophila *are inducible by plant alkaloids and may be important in plant host interactions [45]. In *D. pulex*, differential expression of two Cyp4 genes is associated with resistance to tannic acid and leaf litter [15]. Cyp4C32 expression is much higher in ecotype 1 and Cyp4AP1 shows much higher levels of transcription in ecotype 2, which is exposed to high amounts of leaf litter and polyphenols, and in turn resistant to toxic leaf litters [15]. Interestingly, the CYPs that show differential expression based on ecotype and leaf litter exposure, are the two CYPs that are not found within the *D. pulex *v1.1 genome sequence assembly. The complete sequence of these two CYPs is not available [15].

### Expansion of the CYP 2 and 4 clans

**Figure 4:**
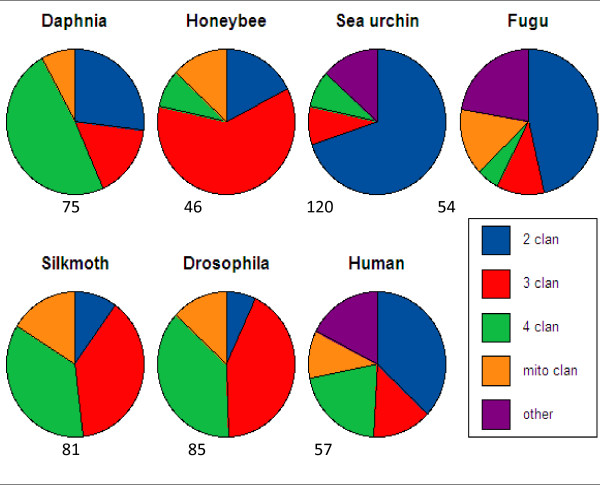
**Comparison of the number of genes in each of the CYP clans from Daphnia, Honeybee, Sea Urchin, Fugu (pufferfish), Silkmoth, Drosophila, and Human genomes**. The data was derived from [22,46-49] and manual curation of the *Daphnia pulex *genome.

A comparison of the number of genes in each of the CYP clans from *D. pulex*, silkmoth (*Bombyx mori*), human (*Homo sapiens*), pufferfish (Fugu rubripes), honeybee (*Apis mellifera*), sea urchin (*Stronglyocentrotus purpuratus*), and fruitfly (*Drosophila melanogaster*) indicates both subtle and demonstrative differences in CYP clan numbers between species (Fig. 4). In general, the protostomes (fruitfly, honeybee, *D. pulex*) have significantly fewer CYP2 clan members than deuterostomes, indicating an expansion of the CYP2 clan in deuterostomes (Fig. 3). Of the protostomes investigated, *D. pulex *has the greatest percentage of CYP2 clan members. The CYP2 clan encompasses approximately 5.5–10% of the total CYPs in most insects with honeybee the exception at 17.4% of its CYPs in the CYP2 clan [22]. In contrast, *D. pulex *has 21 CYPs in the CYP2 clan, nearly double of any sequenced insect (11/132 in *Aedes aegypti*), and *D. pulex's *CYP2 clan members encompass 26.9% of its CYPs, indicating a significant expansion of this clan relative to the insects. No other crustacean has been sequenced, so whether the expansion of the CYP2 clan is typical of crustaceans is unknown.

The CYP4 clan is also slightly expanded in *D. pulex *relative to the insects. There are 38 CYP4 members that encompass 49% of the CYPs in the genome. The CYP4 clan varies from 8.6–42% of the CYP genome in sequenced insects [22]. Excluding the honeybee, which only has 4 CYP4 members, the rest of the insect's CYP4 members vary from 30.7–42% of the total CYPs. Relative increases in CYP2 and CYP4 members in *D. pulex *leaves a relative reduction in the CYP3 clan compared to insects. Only 16.7% (13/78) of the members of the *D. pulex *CYP genome are CYP3 clan members; whereas 38–61% (28–76) of the insect CYPs are CYP3 clan members.

There are several CYP2-clan members similar in structure to other ecdysone metabolizers (CYP18, CYP364 members). However, the formation of juvenile hormone III from methyl farnesoate by CYP15A1 is unnecessary in crustaceans and *D. pulex *in turn lacks the CYP15A1 gene. In addition, D. pulex lacks the CYP303 subfamily members with unknown but putative external sensory development function [5]. Nevertheless, the CYP370 family, which is phylogenetically related to the CYP15 and CYP303 families, has expanded dramatically in *D. pulex*, and this family lacks a specific enzyme with a known function. Based on our current knowledge of CYPs, the expansion of the CYP370 family is probably necessary for responses to environmental stressors such as toxicants, and/or other growth or behavioral stimulators such as plant alkaloid toxins.

### Tandem repeat regions

**Figure 5:**
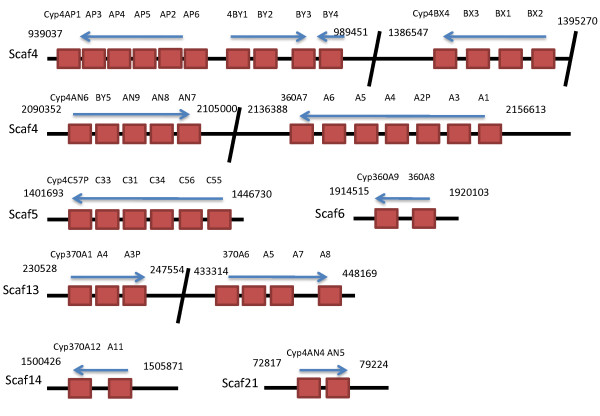
**The Cytochrome P450s are often found in tandem repeats on the genome**. Forty-five of the seventy-seven discovered CYPs are found on tandem repeat regions, and an additional CYP (Cyp4C53; 1481785) is found adjacent to the Cyp4C-rich region on scaffold 5 (not shown). The scaffold numbers are presented on the left hand side of the figure and the positions of each tandem repeat on the scaffold are presented before and after each break in the tandem repeat region. Arrows indicate the direction of the repeat regions, and the names of each CYP are provided above each block that represents a single CYP gene.

Tandem duplicates are genes that are within intron distances of each other, nearly identical (95+% identity), and may harbor some interesting biology. Gene expansion by tandem duplication is common in P450 evolution but the basis for recruitment of a founder gene is not understood. There are several CYP tandem repeat regions in the *D. pulex *genome (Fig. 5). Overall, 45 of the 77 (58%) CYP genes are located within tandem repeats. Twenty-eight of the 37 (76%) CYP4 family members, including Cyp4C53, are found in tandem repeat regions, which explain in part the expansion of the CYP4 clan in *D. pulex*. In contrast, the CYP3 clan has 69% (9/13) of its genes found in tandem repeat regions, including 9/11 of the Cyp360 family members. However, the CYP3 clan is small relative to the insect genomes, thus presence in tandem repeats is not correlated to the size of the clan. Lastly, none of the mitochondrial CYPs, many of which have specific functions in ecdysone synthesis [19], are members of a tandem repeat region (Fig. 4). Expansions of conserved endogenous functions are rare and may be selected against. Conversely, tandem duplications may indicate diversity in exogenous xenobiotic substrates [3].

The CYP2 clan is highly expanded relative to the insects, but only 9 of the 21 CYP2 (43%) clan genes are members of a tandem repeat region. Initially, we thought this indicated that tandem repeat regions had little to do with the expansion of the CYP2 clan in *D. pulex*. However, all nine of the tandemly repeated CYP2 clan members are in the CYP370 family. It is interesting that the rest of the CYP2 clan members are not found in tandem repeat regions, and it is tempting to speculate that most *D. pulex *CYP2 clan members, just as many of the mitochondrial CYPs, have highly specific functions such as ecdysone biosynthesis. Needs for P450s are often met by expansion via tandem duplication leading to gene clusters. This suggests that the CYP370 family expanded while under selective pressure. Which genes expand may be independent of clan or family membership and depend on substrate specificity required to cope with a new xenobiotic stress. The *C. elegans *genome has almost half of its P450s in the CYP2 clan, yet it only has one mitochondrial clan member, CYP44. Insects have expanded the CYP3 clan into the large CYP6 and CYP9 families and several spinoff families. Deuterostomes have expanded CYP2 extensively, while *Trichoplax adhaeren *has only one CYP2 clan member (e_gw1.8.275.1|Triad1 at JGI).

Scaffold 4 is especially rich in tandem repeat regions. It contains 26 of the 44 (59%) CYP genes located in tandem repeats, and 19 of these genes are CYP4 family members. Scaffold 4 contains tandem repeats for CYP4AN, 4AP, 4BX, and 4BY subfamilies. Interestingly, a Cyp4BY subfamily member (Cyp4BY5) is located in the middle of the Cyp4AN subfamily tandem repeat region. This is unusual as most of the genes adjacent to each other in a tandem repeat belong to the same subfamily. As the genes diversify a single gene cluster may contain multiple subfamilies as in the CYP2ABFGST and the CYP4ABXZ gene clusters in mammals [46]. An error could have been made in the annotation of this CYP; however, re-examination of Cyp4BY5 gene provided no evidence of this and this CYP firmly fits in the Cyp4BY subfamily based on identity and phylogenetic status (Fig. 2).

## Conclusion

The cladoceran, *D. pulex *has a wide range of CYPs with the same clans as insects, but with distinct changes in the population of each clan. Of note is the expansion of the newly discovered CYP370 family. Elucidation of the function of the different CYP families and subfamilies will no doubt provide important insight into the ability of *D. pulex *to respond to environmental changes, predator-prey relationships, hormonal changes, plant allelochemicals, and sensory stimuli. Ultimately, the understanding of the evolution of animals will require deciphering the history of the P450s and the *Daphnia *genome may contribute to unraveling molecular aspects of the ecology and physiology of this commonly used crustacean species.

## Methods

### Manual curation

The Joint Genome Institute (JGI)  and wFleaBase  generated gene models for protein-coding genes in *Daphnia pulex *using multiple algorithms. The models were constructed and filtered in order to reduce redundancy based on homology and EST support, which in turn produced the Dappu v1.1 gene builds (July, 2007). The gene models were directly compared to other genomes such as human, mouse, *Drosophila*, zebrafish (*Danio rerio*), Xenopus, and bovine (*Bos taurus*) on the *Daphnia *genome portal. We searched for cytochrome P450 (CYP) gene models using KEGG and KOG pathways in addition to the Advanced Search Options on the JGI *Daphnia pulex *portal, and then manually curated each of the CYPs with the help of the gene models and genome comparisons detailed above. Our manual annotations included corrections to several models based on knowledge of intron-exon boundaries in related genes and BLAST searches as described previously [47], and later assigning gene names based on the homology of *Daphnia *CYPs to other species using defined nomenclature and naming rules for complete genes and pseudogenes [21,46].

### Gross Comparisons and Phylogeny

The different CYP clans and families from *Daphnia pulex *were compared to CYP genomes from human (*Homo sapiens*), honeybee (*Apis mellifera*), fruitfly (*Drosophila melanogaster*), silkmoth (*Bombyx mori*), purple sea urchin (*Stronglyocentrotus purpuratus*), and pufferfish (*Fugu rubripes*) [22,46-49]. In addition, phylogenetic comparisons of the different full length *Daphnia pulex *CYP genes were performed with full length human (*Homo sapiens*), fruitfly (*Drosophila melanogaster*), and honeybee (*Apis mellifera*), and select starlet sea anemone (*Nematostella vectensis*) CYPs. Honeybee, fruitfly, and human CYPs have been manually curated, but the anemone CYPs are only available through GenBank, , or the Nelson cytochrome P450 webpage [49], and have not been curated or officially named yet. This genome was chosen as an ancient, diploblast for comparison to protostome (honeybee, fruitfly, *Daphnia*) and deuterostome (human) CYP genomes.

To construct phylogenetic trees, all of the *Daphnia*, human, fruitfly, honeybee, and anemone CYP amino acid sequences were first aligned using default parameters in ClustalX [50]. Trees were constructed using two methods. First, we used maximum parsimony as implemented in PAUP 4.0b10 [51], a method that minimizes the number of evolutionary events but does not use an explicit substitution model. The parsimony tree was based on a heuristic search with 10 random addition sequence replications and tree-bisection-reconnection branch swapping, with gaps treated as missing data. A 50% majority-rule tree was computed for all equally parsimonious trees.

Next, we constructed several trees using Bayesian inference, a probabilistic model-based method of phylogeny reconstruction that is similar to maximum likelihood but which has substantially reduced computation time. Bayesian trees were constructed with MrBayes version 3.1.2 [52] on a computing cluster provided by the Computational Biology Service Unit of Cornell University . We built trees using the "mixed-model" approach in which the Markov chain Monte Carlo sampler explores nine different fixed-rate amino acid substitution models implemented in MrBayes. We used 4 chains with runs of 2.5 million generations with chains sampled every 100 generations and with a burnin of 10,000 trees; the *WAG *[53] model was selected as the best fitting substitution model by MrBayes. Due to the difficulty in choosing an outgroup for such a diverse and ancient gene family, we elected to present phylogenies with a midpoint rooting in which the root is placed halfway between the two most divergent sequences. Midpoint rooting will accurately determine the root of a phylogeny provided that rates of substitution do not vary across the tree. To examine this assumption of a molecular "clock", we repeated the Bayesian analyses with the added constraint of constant rates of amino acid substitution and rooted the resulting trees with a midpoint rooting. The "clock" constrained trees yielded the same overall topology and root position as the unconstrained analyses, indicating that our application of a midpoint rooting is justified.

### Tandem repeat regions

Automated genome annotation software that relies on alignment with gapping, from BLAST and BLAT thru GeneWise, Exonerate and similar tools, may have problems identifying tandem genes and pseudogenes in areas with highly identical exons [54]. Software may skip over one exon and link to its nearly identical downstream gene model in tandem repeat regions. The *Daphnia pulex *genome appears rich in tandem genes, and CYPs are often found in tandem repeat regions. Tandemgenes, or 'Tandy', software  was used to address this problem, and potential CYPs found in tandem repeat areas were provided by Don Gilbert, Biology, Indiana University . Areas potentially containing tandem repeats were carefully mined and manually curated using the newly generated models, and maps were made of each of the tandem repeat regions.

## Authors' contributions

WSB conceived and coordinated the study, annotated the CYPs, aligned the sequence data, and was the primary author drafting the manuscript. PBM carried out the phylogenetic analysis and helped draft the manuscript. DRN checked the CYP annotations, named the CYPs, provided valuable direction, insight, and expertise on CYP evolution, and helped draft the manuscript. All authors read and approved the final manuscript.

## Supplementary Material

Additional file 1 **Comparison of the number of functional CYP genes in different genomes**. A pdf table comparing the number of functional CYP genes from several different organisms. Click here for file

Additional file 2 **Phylogenetic relationship of the different CYP clans**. Five CYP genomes were subjected to phylogenetic comparisons using MrBayes. An (H) after the CYP name denotes human sequences, a (B) denotes honeybee sequences, a (M) denotes fruitfly sequences, CYP names lacking a letter are *D. pulex *sequences, and anemone sequences are noted with their GenBank protein accession numbers (start with XM). Click here for file

Additional file 3 **Detailed summary of each CYP sequence, its position on the *D. pulex *genome, and other potentially helpful information**. An Excel workbook (xlsx) table of each of the CYP genes and pseudogenes found in the *Daphnia pulex *genome, their nucleotide and amino acid sequences, links to their scaffold position, manual protein identifying number, and comments about the annotation and/or presence of an expressed sequence tag (EST). Click here for file

Additional file 4 **Phylogenetic tree of the mitochondrial CYP clan members**. An (H) after the CYP name denotes human sequences, a (B) denotes honeybee sequences, a (M) denotes fruitfly sequences, CYP names lacking a letter are *D. pulex *sequences, and anemone sequences are noted with their GenBank protein accession numbers (start with XM). Click here for file

Additional file 5 **Phylogenetic tree of the CYP2 clan members**. An (H) after the CYP name denotes human sequences, a (B) denotes honeybee sequences, a (M) denotes fruitfly sequences, CYP names lacking a letter are *D. pulex *sequences, and anemone sequences are noted with their GenBank protein accession numbers (start with XM). Click here for file

Additional file 6 **Phylogenetic tree of the CYP3 clan members**. An (H) after the CYP name denotes human sequences, a (B) denotes honeybee sequences, a (M) denotes fruitfly sequences, CYP names lacking a letter are *D. pulex *sequences, and anemone sequences are noted with their GenBank protein accession numbers (start with XM). Click here for file

Additional file 7 **Phylogenetic tree of the CYP4 clan members**. An (H) after the CYP name denotes human sequences, a (B) denotes honeybee sequences, a (M) denotes fruitfly sequences, CYP names lacking a letter are *D. pulex *sequences, and anemone sequences are noted with their GenBank protein accession numbers (start with XM). Click here for file

## References

[B1] Wei P, Zhang J, Egan-Hafley M, Liang S, Moore DD (2000). The nuclear receptor CAR mediates specific xenobiotic induction of drug metabolism. Nature.

[B2] Kang HS, Angers M, Beak JY, Wu X, Gimble JM, Wada T, Xie W, Collins JB, Grissom SF, Jetten AM (2007). Gene expression profiling reveals a regulatory role for ROR alpha and ROR gamma in phase I and phase II metabolism. Physiol Genomics.

[B3] Li X, Schuler MA, Berenbaum MR (2007). Molecular mechanisms of metabolic resistance to synthetic and natural xenobiotics. Annu Rev Entomol.

[B4] Estabrook RW (2003). A passion for P450s (rememberances of the early history of research on cytochrome P450). Drug Metab Dispos.

[B5] Willingham AT, Keil T (2004). A tissue specific cytochrome P450 required for the structure and function of *Drosophila *sensory organs. Mech Dev.

[B6] Waxman DJ (1988). Interactions of hepatic cytochromes P-450 with steroid hormones: Regioselectivity and stereoselectivity of steroid metabolism and hormonal regulation of rat P-450 enzyme expression. Biochem Pharmcol.

[B7] Waxman DJ, Pampori NA, Ram PA, Agrawal AK, Shapiro BH (1991). Interpulse interval in circulating growth hormone patterns regulates sexually dimorphic expression of hepatic cytochrome P450. Proc Natl Acad Sci USA.

[B8] Muerhoff AS, Griffin KJ, Johnson EF (1994). The peroxisome proliferator-activated receptor mediates the induction of CYP4A6, a cytochrome P450 fatty acid omega-hydroxylase, by clofibric acid. J Biol Chem.

[B9] Luo T, Sakai Y, Wagner E, Dräger UC (2006). Retinoids, eye development, and maturation of visual function. J Neurobiol.

[B10] Sirbu IO, Gresh L, Barra J, Duester G (2005). Shifting boundaries of retinoic acid activity control hindbrain segmental gene expression. Development.

[B11] Tahayato A, Dollé P, Petkovich M (2003). Cyp26C1 encodes a novel retinoic acid-metabolizing enzyme expressed in the hindbrain, inner ear, first branchial arch and tooth buds during murine development. Gene Expr Patterns.

[B12] Maïbèche-Coisne M, Merlin C, François MC, Porcheron P, Jacquin-Joly E (2005). P450 and P450 reductase cDNAs from the moth Mamestra brassicae: cloning and expression patterns in male antennae. Gene.

[B13] Wondji CS, Morgan J, Coetzee M, Hunt RH, Steen K, Black WCt, Hemingway J, Ranson H (2007). Mapping a quantitative trait locus (QTL) conferring pyrethroid resistance in the African malaria vector *Anopheles funestus*. BMC Genomics.

[B14] Fogleman JC, Danielson PB, MacIntyre RJ (1998). The molecular basis of adaptation in Drosophila – The role of cytochrome P450s. Evol Biol.

[B15] David P, Dauphin-Villemant D, Mesneau A, Meyran C (2003). Molecular approach to aquatic environmental bioreporting: differential response to environmental inducers of cytochrome P450 monooxygenase genes in the detritivorous subalpine planktonic Crustacea, *Daphnia pulex*. Mol Ecol.

[B16] Yang Y, Chen S, Wu S, Yue L, Wu Y (2006). Constitutive overexpression of multiple cytochrome P450 genes associated with pyrethroid resistance in *Helicoverpa armigera*. J Econ Entomol.

[B17] Le Goff G, Hilliou F, Siegfried BD, Boundy S, Wajnberg E, Sofer L, Audant P, ffrench-Constant RH, Feyereisen R (2006). Xenobiotic response in Drosophila melanogaster: sex dependence of P450 and GST gene induction. Insect Biochem Mol Biol.

[B18] Claudianos C, Ranson H, Johnson RM, Biswas S, Schuler MA, Berenbaum MR, Feyereisen R, Oakeshott JG (2006). A deficit of detoxification enzymes: pesticide sensitivity and environmental response in the honeybee. Insect Mol Biol.

[B19] Rewitz KF, Rybczynski R, Warren JT, Gilbert LI (2006). The Halloween genes code for cytochrome P450 enzymes mediating synthesis of the insect moulting hormone. Biochem Soc Trans.

[B20] Baldwin WS, LeBlanc GA (1994). Identification of multiple steroid hydroxylases in *Daphnia magna *and their modulation by xenobiotics. Environ Toxicol Chem.

[B21] Nelson DR (2006). Cytochrome P450 nomenclature, 2004. Methods Mol Biol.

[B22] Feyereisen R (2006). Evolution of insect P450. Biochem Soc Trans.

[B23] Rewitz KF, Gilbert LI (2008). Daphnia Halloween genes that encode cytochrome P450s mediating the synthesis of the arthropod molting hormone: evolutionary implications. BMC Evol Biol.

[B24] Rewitz KF, O'Connor MB, Gilbert LI (2007). Molecular evolution of the insect Halloween family of cytochrome P450s: phylogeny, gene organization and functional conservation. Insect Biochem Mol Biol.

[B25] Ono H, Rewitz KF, Shinoda T, Itoyama K, Petryk A, Rybczynski R, Jarcho M, Warren JT, Marques G, Shimell MJ, Gilbert LI, O'Connor MB (2006). *Spook *and *Spookier *code for stage-specific components of the ecdysone biosynthetic pathway in Diptera. Dev Biol.

[B26] Hahn ME (2002). Aryl hydrocarbon receptors: diversity and evolution. Chem Biol Interact.

[B27] Helvig C, Koener JF, Unnithan GC, Feyereisen R (2004). CYP15A1, the cytochrome P450 that catalyzes epoxidation of methyl farnesoate to juvenile hormone III in cockroach corpora allata. Proc Natl Acad Sci USA.

[B28] LeBlanc GA (2007). Crustacean endocrine toxicology: a review. Ecotoxicology.

[B29] Bassett MH, McCarthy JL, Waterman MR, Sliter TJ (1997). Sequenceand developmental expression of Cyp18, a member of a new cytochrome P450 family from *Drosophila*. Mol Cell Endocrinol.

[B30] Strode C, Wondji CS, David JP, Hawkes NJ, Lumjuan N, Nelson DR, Drane DR, Karunaratne SH, Hemingway J, Black WC, Ranson H (2008). Genomic analysis of detoxification genes in the mosquito Aedes aegypti. Insect Biochem Mol Biol.

[B31] Kretschmer XC, Baldwin WS (2005). CAR and PXR: Xenosensors of Endocrine Disrupters?. Chem-Biol Interac.

[B32] Waxman DJ (1999). P450 gene induction by structurally diverse xenochemicals; central role of nuclear receptors CAR, PXR, and PPAR. Arch Biochem Biophys.

[B33] Zhang J, Huang W, Qatanani M, Evans RM, Moore DD (2004). The constitutive androstane receptor and pregnane × receptor function coordinately to prevent bile acid-induced hepatotoxicity. J Biol Chem.

[B34] Masuyama H, Hiramatsu Y, Mizutani Y, Inoshita H, Kudo T (2001). The expression of pregnane × receptor and its target gene, cytochrome P450 3A1 in perinatal mouse. Mol Cell Endocrinol.

[B35] Waxman DJ, Ko A, Walsh C (1983). Regioselectivity and stereoselectivity of androgen hydroxylations catalyzed by cytochrome P-450 isozymes purified from phenobabital-induced rat liver. J Biol Chem.

[B36] Acevedo R, Villanueva H, Parnell PG, Chapman LM, Gimenez T, Gray SL, Baldwin WS (2005). The contribution of hepatic steroid metabolism to serum estradiol and estriol concentrations in nonylphenol treated MMTVneu mice and its potential effects on breast cancer incidence and latency. J Appl Toxicol.

[B37] Simpson AECM (1997). The cytochrome P450 4 family. Gen Pharmacol.

[B38] Ono H, Ozaka K, Yoshikawa H (2005). Identification of cytochrome P450 and glutathione S-transferase genes preferentially expressed in chemosensory organs of the swallowtail butterflyl, *Pailio xuthus *L. Insect Biochem Mol Biol.

[B39] Bradfield JY, Lee YH, Keeley LL (1991). Cytochrome P450 family 4 in a cockroach: molecular cloning and regulation by regulation by hypertrehalosemic hormone. Proc Natl Acad Sci USA.

[B40] Davies L, Williams DR, Aguiar-Santana IA, Pedersen J, Turner PC, Rees HH (2006). Expression and down-regulation of cytochrome P450 genes of the CYP4 family by ecdysteroid agonists in *Spodoptera littoralis *and *Drosophila melanogaster*. Insect Biochem Mol Biol.

[B41] Zhu F, Liu N (2007). Differential expression of CYP6A5 and CYP6A5v2 in pyrethroid-resistant house flies, *Musca domestica*. Arch Insect Biochem Physiol.

[B42] Joußen N, Heckel DG, Haas M, Schuphan I, Schmidt B (2007). Metabolism of imidacloprid and DDT by P450 CYP6G1 expressed in cell cultures of *Nicotiana tabacum *suggests detoxification of these insecticides in Cyp6g1-overexpressing strains of Drosophila melanogaster, leading to resistance. Pest Manag Sci.

[B43] Scharf ME, Parimi S, Meinke LJ, Chandler LD, Siegfried BD (2001). Expression and induction of three family 4 cytochrome P450 (CYP4)* genes identified from insecticide-resistant and susceptible western corn rootworms, *Diabrotica virgifera virgifera*. Insect Mol Biol.

[B44] Daborn PJ, Yen JL, Bogwitz MR, Le Goff G, Feil E, Jeffers S, Tijet N, Perry T, Heckel D, Batterham P, Feyereisen R, Wilson TG, ffrench-Constant RH (2002). A single P450 allele associated with insecticide resistance in Drosophila. Science.

[B45] Danielson PB, Foster JLM, NcNahill MM, Smith MK, Fogleman JC (1998). Induction by alkaloids and phenobarbital of family 4 P450s in *Drosophila*: evidence for host plant utilization. Mol Gen Genet.

[B46] Nelson DM, Zeldin DC, Hoffman SMG, Maltais LJ, Wain HM, Nebert DW (2004). Comparison of cytochrome P450 (CYP) genes from the mouse and human genomes, including nomenclature recommendations for genes, pseudogenes and alternative splice variants. Pharmacogenetics.

[B47] Nelson DR (2003). Comparison of P450s from human and fugu: 420 million years of vertebrate P450 evolution. Arch Biochem Biophys.

[B48] Goldstone JV, Hamdoun A, Cole BJ, Howard-Ashby M, Nebert DW, Scally M, Dean M, Epel D, Hahn ME, Stegeman J (2006). The chemical defensome: Environmental sensing and response genes in the *Strongylocentrotus purpuratus *genome. Dev Biol.

[B49] Nelson DR Cytochrome P450 Homepage. http://drnelson.utmem.edu/CytochromeP450.html.

[B50] Thompson JD, Gibson TJ, Plewniak F, Jeanmougin F, Higgins DG (1997). The clustalx windows interface: flexible strategies for multiple sequence alignment aided by quality analysis tools. Nucleic Acids Res.

[B51] Swofford DL (2001). PAUP*: phylogenetic analysis using parsimony (*and other methods), version 4.0b10.

[B52] Ronquist F, Huelsenbeck JP (2003). MrBayes 3: Bayesian phylogenetic inference under mixed models. Bioinformatics.

[B53] Whelan S, Goldman N (2001). A general empirical model of protein evolution derived from multiple protein families using a maximum-likelihood approach. Mol Biol Evol.

[B54] Nelson DR (2004). Frankenstein genes, or the Mad Magazine version of the human pseudogenome. Human Genomics.

