# Supplementary material for: The cytochrome P450 (CYP) gene superfamily in Daphnia pulex
Source: BMC Genomics. 2009 Apr 21;10:169. doi: 10.1186/1471-2164-10-169 (PMC2678163; doi:10.1186/1471-2164-10-169)
Supplement: Additional file 1 — Comparison of the number of functional CYP genes in different genomes. A pdf table comparing the number of functional CYP genes from several different organisms. [file 1471-2164-10-169-S1.pdf]

Additional File 1: Comparison of the number of functional CYP genes in different genomes.

Species	No. of genes[a]
Daphnia pulex (waterflea)	75[b]
Homo sapiens (human)	57
Mus musculus (mouse)	102
Stronglyocentrotus purpuratus (sea urchin)	120
Apis mellifera (honeybee)	46
Drosophila melanogaster (fruitfly)	83
Fugu ruppripes (pufferfish)	54
Anopholes gambiae (mosquito)	106
Bombyx mori (silkmoth)	81

[a]Based on estimates as of November 2007.

[b]*Daphnia pulex* has an additional three pseudogenes.
