# Supplementary figures and images for: The cytochrome P450 (CYP) gene superfamily in Daphnia pulex
Source: BMC Genomics. 2009 Apr 21;10:169. doi: 10.1186/1471-2164-10-169 (PMC2678163; doi:10.1186/1471-2164-10-169)

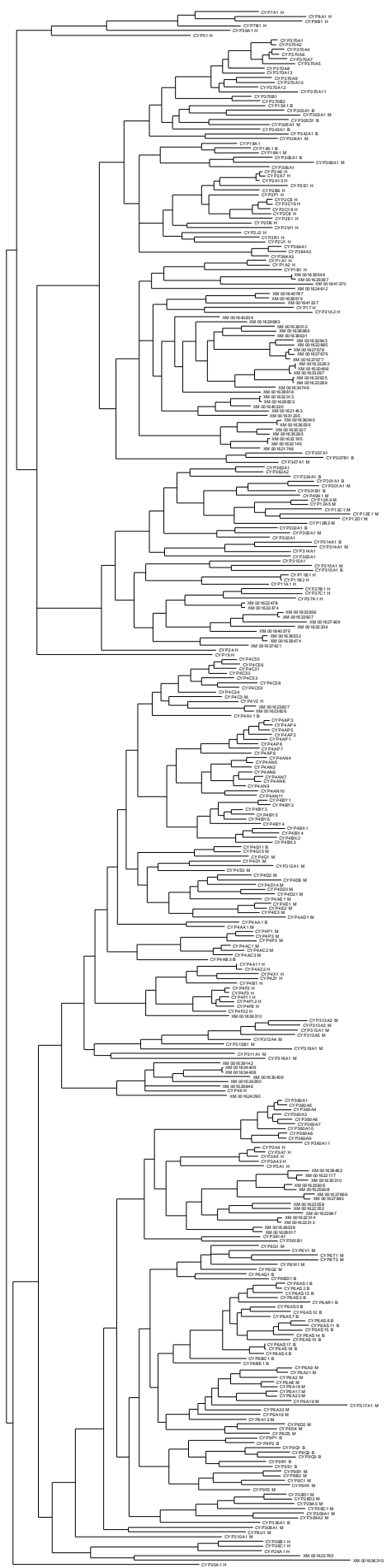

0.1 changes

Supplement: Additional file 2 — Phylogenetic relationship of the different CYP clans. Five CYP genomes were subjected to phylogenetic comparisons using MrBayes. An (H) after the CYP name denotes human sequences, a (B) denotes honeybee sequences, a (M) denotes fruitfly sequences, CYP names lacking a letter are D. pulex sequences, and anemone sequences are noted with their GenBank protein accession numbers (start with XM). [file 1471-2164-10-169-S2.pdf]

# Mitochondrial

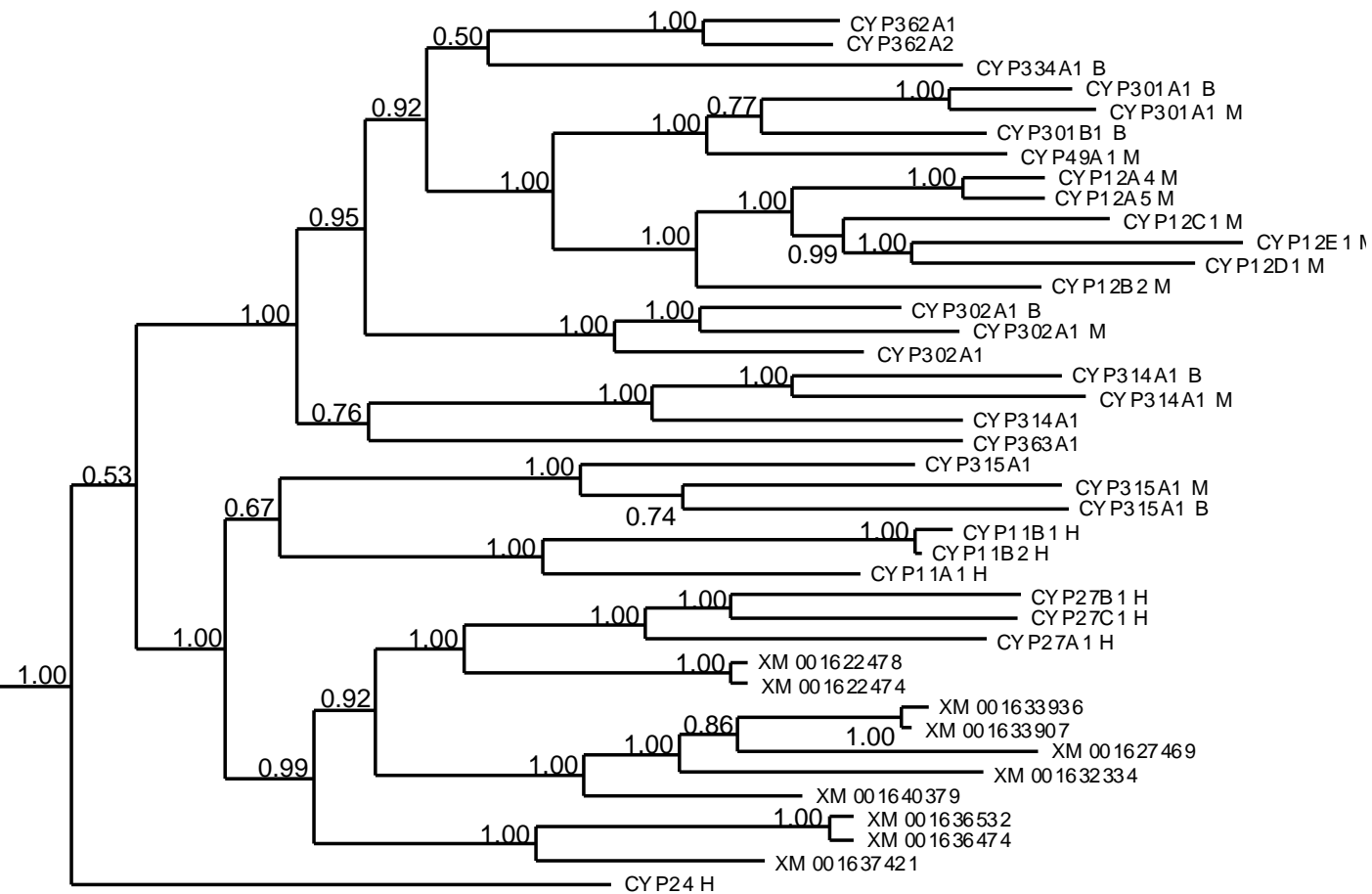

Supplement: Additional file 4 — Phylogenetic tree of the mitochondrial CYP clan members. An (H) after the CYP name denotes human sequences, a (B) denotes honeybee sequences, a (M) denotes fruitfly sequences, CYP names lacking a letter are D. pulex sequences, and anemone sequences are noted with their GenBank protein accession numbers (start with XM). [file 1471-2164-10-169-S4.pdf]

# CYP2

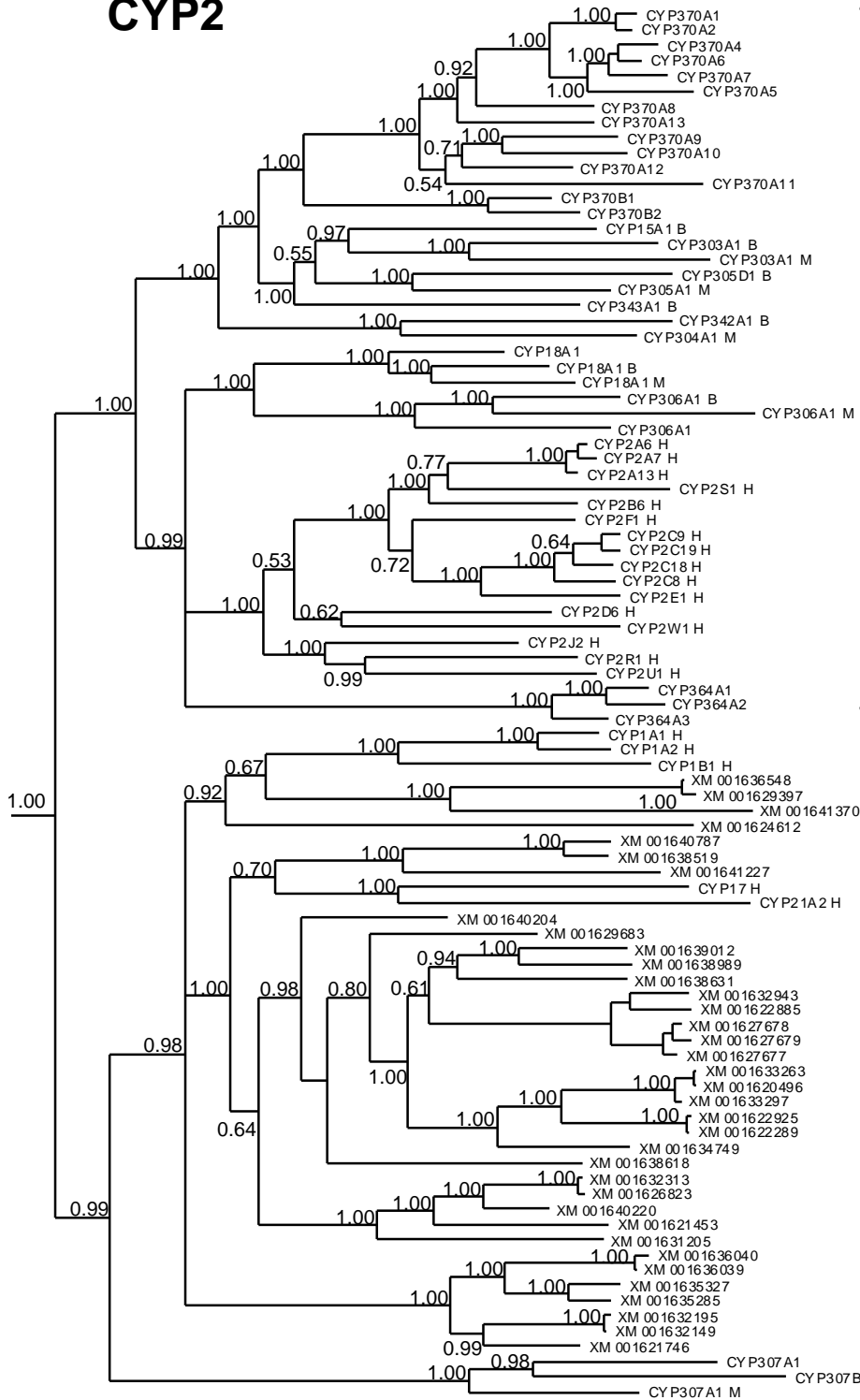

## CYP2

*Daphnia*  
Honeybee  
*Drosophila*  
Human

## CYP2

Human  
Anemone

## CYP307

*Daphnia*  
Honeybee  
*Drosophila*

Supplement: Additional file 5 — Phylogenetic tree of the CYP2 clan members. An (H) after the CYP name denotes human sequences, a (B) denotes honeybee sequences, a (M) denotes fruitfly sequences, CYP names lacking a letter are D. pulex sequences, and anemone sequences are noted with their GenBank protein accession numbers (start with XM). [file 1471-2164-10-169-S5.pdf]

# CYP4

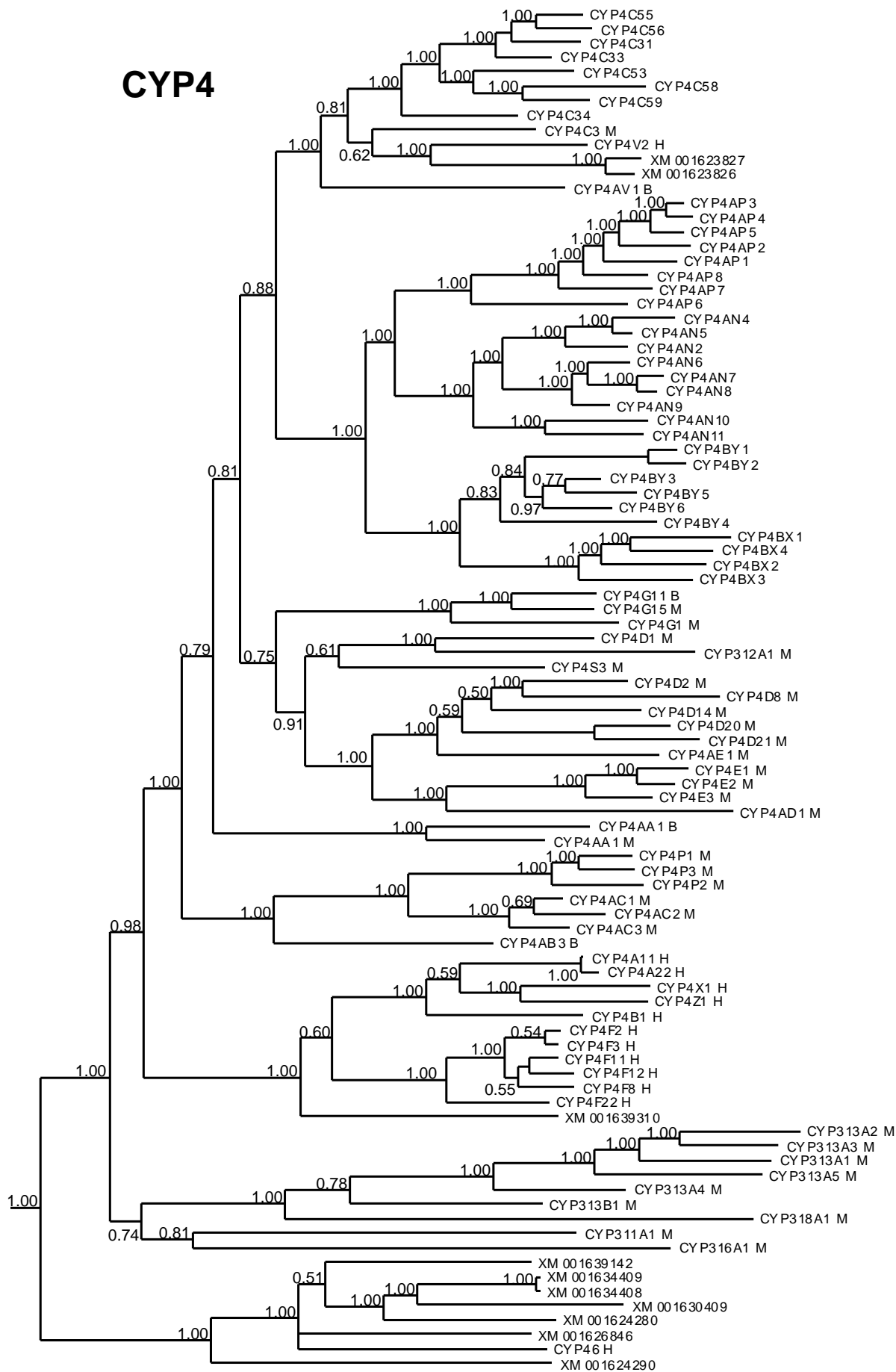

Supplement: Additional file 7 — Phylogenetic tree of the CYP4 clan members. An (H) after the CYP name denotes human sequences, a (B) denotes honeybee sequences, a (M) denotes fruitfly sequences, CYP names lacking a letter are D. pulex sequences, and anemone sequences are noted with their GenBank protein accession numbers (start with XM). [file 1471-2164-10-169-S7.pdf]
