# Supplementary material for: The cytochrome P450 (CYP) gene superfamily in Daphnia pulex
Source: BMC Genomics. 2009 Apr 21;10:169. doi: 10.1186/1471-2164-10-169 (PMC2678163; doi:10.1186/1471-2164-10-169)
Supplement: Additional file 6 — Phylogenetic tree of the CYP3 clan members. An (H) after the CYP name denotes human sequences, a (B) denotes honeybee sequences, a (M) denotes fruitfly sequences, CYP names lacking a letter are D. pulex sequences, and anemone sequences are noted with their GenBank protein accession numbers (start with XM). [file 1471-2164-10-169-S6.pdf]

CYP3

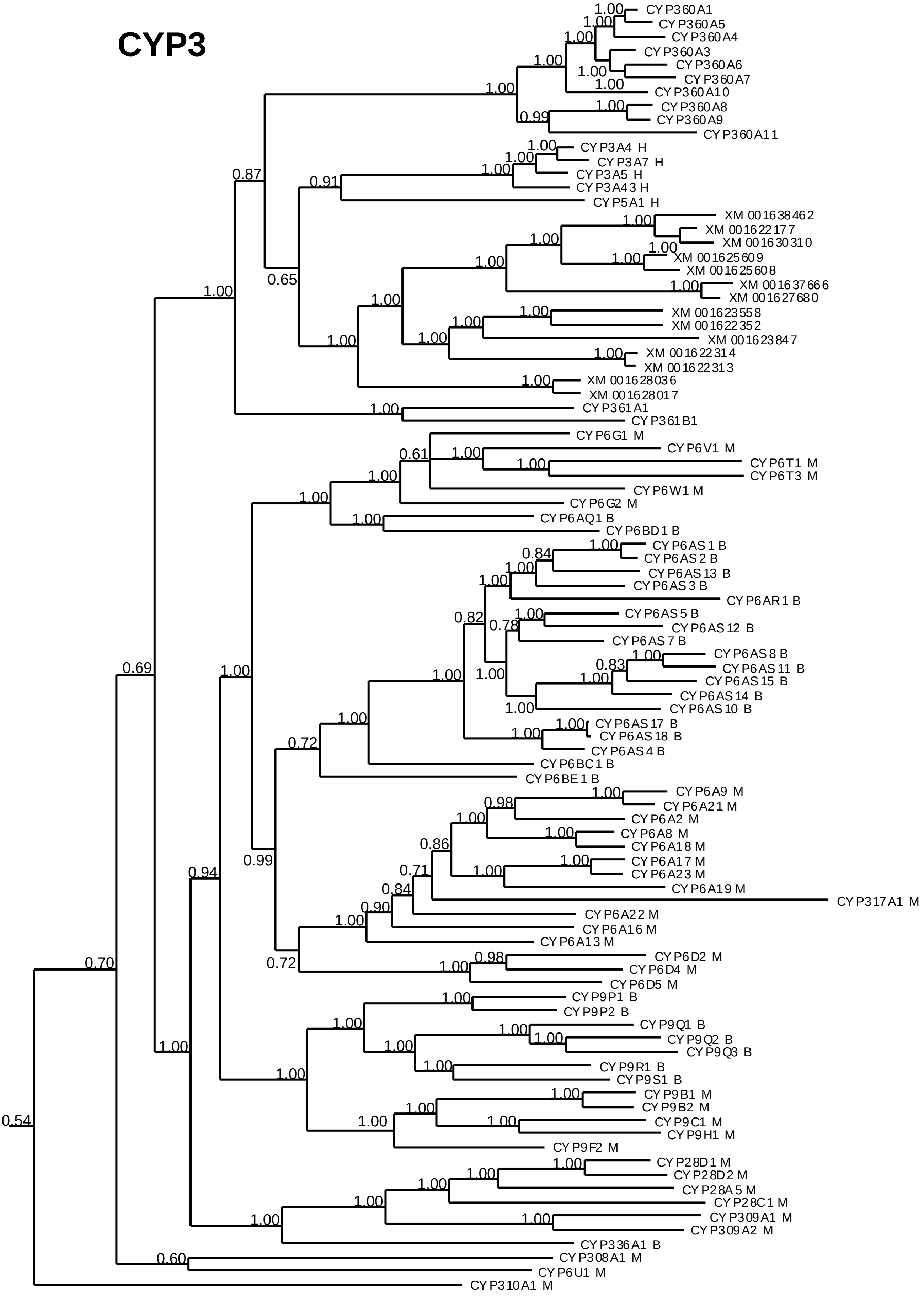
CYP360A1
CYP360A5
CYP360A4
CYP360A3
CYP360A6
CYP360A7
CYP360A10
CYP360A8
CYP360A9
CYP360A11
CYP3A4 H
CYP3A7 H
CYP3A5 H
CYP3A43 H
CYP5A1 H
XM 001638462
XM 001622177
XM 001630310
XM 001625609
XM 001625608
XM 001637666
XM 001627680
XM 001623558
XM 001622352
XM 001623847
XM 001622314
XM 001622313
XM 001628036
XM 001628017
CYP361A1
CYP361B1
CYP6G1 M
CYP6V1 M
CYP6T1 M
CYP6T3 M
CYP6W1 M
CYP6G2 M
CYP6AQ1 B
CYP6BD1 B
CYP6AS1 B
CYP6AS2 B
CYP6AS13 B
CYP6AS3 B
CYP6AR1 B
CYP6AS5 B
CYP6AS12 B
CYP6AS7 B
CYP6AS8 B
CYP6AS11 B
CYP6AS15 B
CYP6AS14 B
CYP6AS10 B
CYP6AS17 B
CYP6AS18 B
CYP6AS4 B
CYP6BC1 B
CYP6BE1 B
CYP6A9 M
CYP6A21 M
CYP6A2 M
CYP6A8 M
CYP6A18 M
CYP6A17 M
CYP6A23 M
CYP6A19 M
CYP317A1 M
CYP6A22 M
CYP6A16 M
CYP6A13 M
CYP6D2 M
CYP6D4 M
CYP6D5 M
CYP9P1 B
CYP9P2 B
CYP9Q1 B
CYP9Q2 B
CYP9Q3 B
CYP9R1 B
CYP9S1 B
CYP9B1 M
CYP9B2 M
CYP9C1 M
CYP9H1 M
CYP9F2 M
CYP28D1 M
CYP28D2 M
CYP28A5 M
CYP28C1 M
CYP309A1 M
CYP309A2 M
CYP336A1 B
CYP308A1 M
CYP6U1 M
CYP310A1 M
